# The causal effect of red blood cell folate on genome-wide methylation in cord blood: a Mendelian randomization approach

**DOI:** 10.1186/1471-2105-14-353

**Published:** 2013-12-04

**Authors:** Alexandra M Binder, Karin B Michels

**Affiliations:** 1 Department of Epidemiology, Harvard School of Public Health, Boston, MA, USA; 2 Obstetrics and Gynecology Epidemiology Center, Department of Obstetrics and Gynecology, Brigham and Women’s Hospital, Harvard Medical School, Boston, MA, USA

**Keywords:** Mendelian randomization, DNA methylation, Folate, Epigenomic epidemiology

## Abstract

**Background:**

Investigation of the biological mechanism by which folate acts to affect fetal development can inform appraisal of expected benefits and risk management. This research is ethically imperative given the ubiquity of folic acid fortified products in the US. Considering that folate is an essential component in the one-carbon metabolism pathway that provides methyl groups for DNA methylation, epigenetic modifications provide a putative molecular mechanism mediating the effect of folic acid supplementation on neonatal and pediatric outcomes.

**Results:**

In this study we use a Mendelian Randomization Unnecessary approach to assess the effect of red blood cell (RBC) folate on genome-wide DNA methylation in cord blood. Site-specific CpG methylation within the proximal promoter regions of approximately 14,500 genes was analyzed using the Illumina Infinium Human Methylation27 Bead Chip for 50 infants from the Epigenetic Birth Cohort at Brigham and Women’s Hospital in Boston. Using methylenetetrahydrofolate reductase genotype as the instrument, the Mendelian Randomization approach identified 7 CpG loci with a significant (mostly positive) association between RBC folate and methylation level. Among the genes in closest proximity to this significant subset of CpG loci, several enriched biologic processes were involved in nucleic acid transport and metabolic processing. Compared to the standard ordinary least squares regression method, our estimates were demonstrated to be more robust to unmeasured confounding.

**Conclusions:**

To the authors’ knowledge, this is the largest genome-wide analysis of the effects of folate on methylation pattern, and the first to employ Mendelian Randomization to assess the effects of an exposure on epigenetic modifications. These results can help guide future analyses of the causal effects of periconceptional folate levels on candidate pathways.

## Background

Identification of the protective effect of periconceptional folic acid supplementation against neural tube defects in neonates led to the mandated fortification of flours and other grain products in several countries [1-4]. In addition to the prevention of neural tube defects, folic acid supplementation has been associated with decreased risk of other congenital malformations, such as heart defects and oral clefts [5-8]. Despite these benefits, concern has been raised to possible adverse effects. In mouse models, maternal methyl donor supplementation was associated with increased risk of allergic airway disease [9,10]. However, subsequent human studies of possible detrimental effects have been relatively inconclusive [11-14]. An understanding of the biological mechanism by which folate acts to affect fetal development can inform appraisal of expected benefits and risk management, and is ethically imperative due to the ubiquity of fortified foods. Folate, an essential vitamin that can be obtained from diet and synthetic supplements, is an important component in the one-carbon metabolism that frees up methyl goups for DNA methylation. Thus, epigenetic modifications provide a putative molecular mechanism mediating the effect of folic acid supplementation on neonatal and pediatric outcomes. Prior observational studies have reported inconsistent associations between maternal folic acid supplementation and maternal folate levels with infant DNA methylation, specifically among imprinted genes [15-17]. One difficulty when studying the association between folic acid supplementation and DNA methylation is the possibility of effects being obfuscated by the influence of diet on total maternal folate levels contributing to dose misclassification. Analyses of the effects of folate levels may also be biased due to the unmeasured confounding by multifaceted environmental exposures associated with socio-economic status that may also influence epigenomic profile. Therefore new approaches are necessary to consistently estimate the causal effect of folate on DNA methylation.

**Figure 1:**
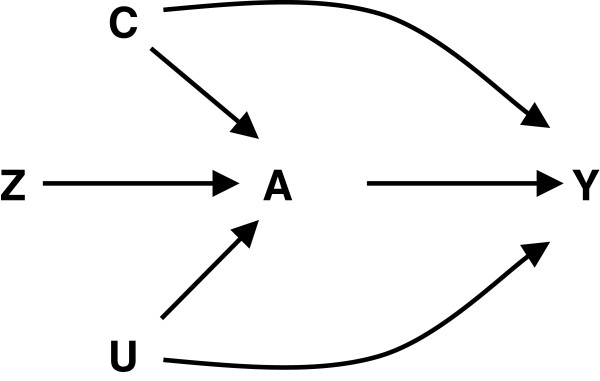
**The structure of an observation study to assess the association between exposure (A) and outcome (Y) in the presence of both measured (C) and unmeasured confounding (U); Z represents a possible instrument for an instrumental variable analysis.** In this study, A represents log transformed RBC folate, the outcome Y is site-specific methylation, C is intention to conceive, our surrogate for confounding by periconceptional behaviors, and our instrument Z is MTHFR genotype modeled additively.

Standard methods to control for confounding in observational studies include matching, restriction, stratification, regression analyses, inverse probability weighting, and g-estimation. However, these techniques are limited by their capacity to only adjust for observed confounders, and rely on the unverifiable condition of no unmeasured confounding to estimate causal effects. Instrumental variable analysis, a conventional method in econometrics that has more recently been applied to public health research, provides a means to adjust for all confounders without exposure randomization. The application of instrumental variable analysis has been reviewed elsewhere [18-20]. Generally, instrumental variable analysis requires a variable (instrument) that has a causal effect on the exposure and only affects the outcome of interest through the exposure, and for which there is no common cause of the instrument and outcome (Figure 1). In addition to these three assumptions, to identify the causal effect we must make a fourth assumption concerning the nature of the influence of the exposure on the outcome [20]. Either we must posit a constant treatment effect, which is implicitly assumed by the two-stage least squares method outlined below, or no effect modification by the instrument on the exposure-outcome association [20]. The two-stage least squares (2SLS) method for instrumental variable estimation for a continuous outcome takes the form:

(1) $$A=\mathrm{\alpha Z}+\epsilon_{a}$$

(2) Y=βA^+ϵy

Where Z is our instrument, A is our exposure of interest, and Y is our outcome. In economic literature, Z is referred to the exogenous variable, i.e. explained by variables outside the model, and A is the endogenous variable, explained by other variable in the model. In the first stage (1), the exposure is regressed on the instruments. The second stage (2) regresses the outcome on the fitted values ( A^) from the first stage. Given the instrument Z meets the conditions outlined above, the parameter estimate from fitting model (2) will provide a consistent estimate of the causal effect of our exposure on our outcome. Similar to the other techniques to control for confounding in observation studies, these assumptions are unverifiable, but reasonable when the instrument is a genetic polymorphism. Using a genetic variant as the instrument, also known as Mendelian Randomization, is an appealing approach to establish temporality and due to the lack of common causes of the instrument and the outcome aside from population stratification [21-23]. Although the instrumental variable estimate will be asymptotically unbiased, in finite samples the instrumental variable estimates will be biased towards the observed confounded association. This bias arises because the true relationship between the instrument and the exposure in the first stage of the analysis is unknown and it must be estimated, resulting in model over-fitting. The magnitude of this bias depends on the strength of the association between the instrument and the exposure [19,24]. Weak instrument bias, which is often a concern for Mendelian Randomization studies, can be minimized and precision increased by including measured confounders in the two-stage analysis [25].

Using common methylenetetrahydrofolate reductase (MTHFR) polymorphisms as our instrument, Mendelian Randomization provides one method to assess the causal effect of maternal folate on epigenetic profile. MTHFR catalyzes the synthesis of 5-methylTHF, which is the coenzyme required for homocysteine remethylation to methionine, the precursor for the DNA methylating agent *S*-adenosylmethionine. Two common polymorphism, C677T and A1298C, are associated with reduced MTHFR enzymatic activity, resulting in higher homocysteine levels [26-28]. In this study we used a Mendelian Randomization approach to assess the effect of red blood cell (RBC) folate on genome-wide DNA methylation in cord blood. The mother’s MTHFR genotype was utilized as our instrument, given the efficiency of maternal folate metabolism would be expected to modify developmental exposure. A long-term measure of folate intake, RBC folate has been demonstrated to be responsive and sensitive to inter-individual differences in controlled folate intake [29-31]. In a prior study of the association between of maternal and newborn folate status, early maternal plasma folate (~18 weeks) and self-reported folic acid supplementation were found to be significantly associated with cord blood folate levels [32]. In our study population of 50 newborns, site-specific CpG methylation within the proximal promoter regions of approximately 14,500 genes was analyzed using the Illumina Infinium Human Methylation27 Bead Chip. With the Mendelian Randomization approach we were able to identify the causal effects of folate on epigenetic modifications that would have been substantially biased given a standard regression analysis. The possible utility of Mendelian Randomization to investigate the causal structure of disease etiology mediated by epigenetic modifications in observational studies has been discussed by others [33-36], but this is the first study to apply this approach.

## Methods

### The epigenetic birth cohort

Data and biospecimens were collected between June 2007 and June 2009 on the labor and delivery floor of the Department of Obstetrics, Gynecology and Reproductive Biology at Brigham and Women’s Hospital in Boston as previously described [37]. Briefly, pregnant women were invited to participate in our study, and 1941 completed a questionnaire, and agreed to donate placenta and cord blood samples. Maternal and cord blood collected from the base of the umbilical cord was stored in EDTA tubes. Blood was processed immediately and buffy coats were stored at -80°C. From this cohort, a subset of 50 cord blood samples were analyzed for genome-wide DNA methylation associated with RBC folate. The study protocol was approved by the Institutional Review Board of the Brigham and Women’s Hospital.

### Genome-wide methylation analysis

For the current study, 1 μg of cord buffy coat DNA from each of the 50 individuals was processed at the USC Epigenome Center (Los Angeles, CA, USA) as previously described [38]. For comprehensive analysis of genome-wide methylation, the Illumina Infinium Human Methylation27 Bead Chip was used to simultaneously interrogate methylation at 27,578 CpG sites, spanning 14,495 RefSeq genes. On average, the Infinium array targets 2 CpG sites per gene, with higher coverage (3–20 CpGs) for cancer-related and imprinted genes. Data was assembled at the Epigenome Center by converting fluorescence intensities from methylated (M) and unmethylated (U) alleles to methylation level given by β = (M)/(M + U + 100). If signal intensity was not significantly different from background measurements, the β-value was recorded “NA.” The statistical analysis was restricted to autosomal CpGs with unique probe target sequences. The 50-mer oligonucleotide probes were removed from further analysis if the probe had (a) cross-reactive target(s) with at least 40 matching bases, at least 90% identity, end-nucleotide match, and gapless sequence alignment against the target sequence [39]. Further restriction to CpG sites with no missing data reduced the data analysis from 23,682 to 23,264 autosomal CpGs. Methylation level for these loci was square-root arcsine transformed to stabilize the variance. Transformed loci that were not normally distributed at the 0.05 α-level were then removed from the dataset (Shapiro-Wilk test). In total, 16,989 loci were analyzed in subsequent statistical analyses.

### Genotyping

Genotyping of the maternal MTHFR C677T (rs1801133) and A1298C (rs1801131) SNPs was performed on the ABI PRISMs 7300 Real-Time PCR System (Applied Biosystems, Foster City, CA, USA). Primers and probes were ordered from TaqMan® SNP Genotyping Assays (Applied Biosystems) MTHFR C677T (assay ID: C___1202883) and A1298C (assay ID: C____850486). The PCR was performed in 25 ul, with each reaction containing 25 ng gDNA, 1.25 ul of 20× Assay Mix, 12.5 ul of TaqMan genotyping master mix and *q.s.* with PCR grade water. Cycling conditions were as follows: 50°C for 2 min, 95°C for 10 min, and 40 cycles of 92°C for 15 s and 60°C for 1 min. After the amplification, plates were scanned by the ABI PRISMs 7500 PCR system to determine genotypes by allelic discrimination. Hardy-Weinberg assumptions were assessed using a Chi-square test.

### Red blood cell folate

Cord blood RBC folate was measured on the Roche E Modular system (Roche Diagnostics, Indianapolis, IN) in the laboratory of Dr. Nader Rifai at Children”s Hopital in Boston, MA, USA. Serum and plasma folate are sensitive to day-to-day variation in intake, reflecting short-term diet [40,41]. We chose to assay RBC folate given is more stable marker of long-term patterns [42]. Red blood cells were first lysed with ascorbic acid, and folate was then measured on the hemolysate. Hemoglobin was also measured on this hemolysate to standardize RBC folate per gram of hemoglobin. The sample was treated with monothioglycerol and sodium hydroxide to release the folate from endogenous binding proteins, and incubated with a ruthenium labeled folate binding protein, forming a folate complex. Biotinylated folate and streptavidin-coated magnetic microparticles were then added to the reaction mixture. Ruthenium labeled biotin complexes bound to the magnetic microparticles, and unbound reagents and sample were washed away. A chemiluminescent reaction was electrically stimulated to generate light, the intensity being indirectly proportional to the amount of folate present in the sample. This assay is approved by the Food and Drug Administration for clinical use. The lowest detection limit of this assay is 0.6 ng/mL, and the day-to-day imprecision values at concentrations of 7.6, 14.3 and 19.2 ng/mL are 3.9, 3.1 and 2.0%, respectively. The normal range is 3.1 to 17.5 ng/mL. The positively skewed RBC folate measurements were log transformed for subsequent statistical analyses. In this cohort, log RBC folate ranged from 6.13 to 7.54 log (ng/mL), with a median level of 6.664 log (ng/mL).

### Statistical analysis

To estimate the effect of RBC folate on DNA methylation, we exploited Mendelian Randomization methods using the two-stage least squares (TSLS) approach. In the first stage, log transformed RBC folate was regressed against MTHFR genotype modeled additively. Predicted values from this first stage were then used to model square-root arcsine transformed methylation levels. An indicator for whether conception was planned, a putative confounder, was included in both stages to increase precision and decrease weak instrument bias. The effect of RBC folate on methylation level using the TSLS approach was estimated for each CpG locus independently using the AER package in R [43]. Among the sites that had significant changes in methylation at the 0.05 α-level, the effect of log transformed RBC folate on untransformed β-values was estimated using TSLS. While this yielded valid, more interpretable parameter estimates, inference was biased due to the violation of the ordinary least squares normality assumptions. Therefore robust 95% confidence intervals for these estimates were generated by bootstrapping 1000 replicates. The UCSC Genome Browser database was used to characterize the location of the significant CpG sites in relation to annotated features and CpG islands [44]. Functional enrichment among the genes in closest proximity to the significant sites compared to all Gene Ontology (GO) annotated genes represented on the Illumina microarray was evaluated based on biological process using the GOstats package provided by Bioconductor [45]. Overrepresentation in the data set was assessed using a conditional Hypergeometric test, which considers the relationships between the GO terms and conditions on the significant child terms.

The association between log transformed RBC folate and methylation level for each CpG site, adjusting for whether conception was planned, was also assessed using ordinary least squares. Only conception intention was controlled for in these standard regression models so that the interpretation of the parameter estimates would be comparable to the effect estimates obtained using the TSLS method. The 10 most significant loci identified using the ordinary least squares approach were compared to the significant TSLS sites. Global disparities in parameter estimates between the Mendelian Randomization and standard approach were visualized with a scatterplot. Pearson’s correlation was used to formally assess the relationship between the parameter estimates from the two methods. To assess the ability of Mendelian Randomization to estimate the true causal effect in the presence of unmeasured confounding compared to a standard analysis, 10,000 datasets were simulated with varying degrees of unmeasured confounding and effects sizes. For each of these simulations, 50 individual exposure (*x*_*i*_) and outcome (*y*_*i*_) values were generated as shown below:

xi=α^0+α^1genotypei+α^2conceptioni+αcui+exiyi=β^0+β^1logRBCfolatei+β^2conceptioni+βcui+eyi

$$e_{\mathrm{xi}},e_{\mathrm{yi}},u_{i}∼N\left(0,1\right)$$

In both the exposure and outcome models, the confounder *u*_*i*_ and error term were independently normally distributed. Parameters α^ and β^ estimated from each significant TSLS model were assumed to be the true population values, and were used to create the simulated outcome and exposure values. Genotype, planned vs. unplanned conception, and RBC folate corresponded to the observed values of each individual. The distribution of effect estimates were assessed among the 10,000 simulated datasets using Mendelian Randomization and ordinary least squares approaches adjusting for conception, for *α*_*c*_ = *β*_*c*_ = 0.1 and 0.2. All statistical analyses were conducted using R version 2.15.2.

### Pyrosequencing

Four of CpG loci indicated to be significantly associated with RBC folate by the MR approach were selected for verification by pyrosequencing, chosen to reflect varying effect sizes. These regions were in proximity to the transcription start site of four genes: *AIRE, GPR12, OBFC2B,* and *SMG6*. Pyrosequencing of the regions surrounding these significant loci was performed by a commercial service (EpigenDx, Worcester, MA) on a subset of 38 samples in duplicate. EpigenDx performed the bisulfite conversion, designed the assays, and pyrosequenced the regions, including high and low methylation controls. The association between methylation and folate level at each locus interrogated by pyrosequencing was estimated by MR as previously described. Regional changes were visualized by a loess curve of the site-specific estimates plotted against genomic location.

## Results and discussion

The global minor allele frequencies estimated in the 1000 Genome phase 1 population are approximately 33% for C677T (rs1801133) and 23% for the A1298C (rs1801131). Both SNPs were in Hardy-Weinberg equilibrium among our study population (p = 0.129; p = 0.433). Except for a very rare cis 677 T/1298C haplotype found in some parts of the United Kingdom and Canada, most 677 T and 1298C alleles are associated with 1298A and 677C alleles, respectively [46]. Thus modeling MTHFR genotype additively corresponded to modeling the risk associated with having zero, one, or two common trans MTHFR variants. Modeling the number of variants co-dominantly did not significantly explain more variation in log transformed RBC folate than the additive model (F = 1.335, p = 0.254).

For each variant MTHFR allele, there was significant increase in log RBC folate, adjusting for conception intention (F = 4.876, p = 0.0321). Elevated RBC folate associated with MTHFR polymorphisms has been observed in prior studies that also utilized radio assay techniques [47-50]. Intent to conceive was included in subsequent analyses as a surrogate for periconceptional behaviors that may influence both RBC folate and methylation level. Using maternal MTHFR genotype modeled additively as the instrument, the TSLS approach identified 7 CpG loci with a significant association between log RBC folate and transformed methylation level (Table 1). Of these significant loci, 5 were within CpG islands, and two were associated with a CpG island shore. A majority of these loci were within a 5′ UTR. One CpG locus was within an enhancer, and another was promoter associated. A one unit increase in log RBC folate was associated with an increase in methylation level for most of this subset. Given the levels of log RBC folate in this population of healthy neonates ranged from 6.13 to 7.54 log(ng/mL), a one unit change in log RBC folate was considered to be within reasonable exposure variation. Among the genes in closest proximity to this significant subset of CpG loci, the regulation of dephosphorylation and the degradation of prematurely truncated transcripts were the most significantly enriched biological processes (Table 2). Several enriched processes were involved in nucleic acid transport and metabolic processing. While we did not adjust for the possible methylation variation mediated by the impact of folate on cell type distribution, only one of the significant loci was associated with autoimmune response, indicating cell type fluctuations were not a major determinant of the strongest associations.

**Table 1:** Genes in proximity to loci with a significant change in methylation levels per one unit change in log (RBC folate)

**Symbol**	**Chr**	**UCSC annotated features**	**CpG island**	**Δ β-value**	**Bootstrap 95% CI**
OBFC2B	12	Promoter associated	Shore	0.386	(0.227, 0.551)
GPR75; LOC100302652	2	5′UTR; 1st Exon; Enhancer	Island	0.349	(0.184, 0.515)
AIRE	21	Within 100 bp of TSS	Island	0.267	(0.094, 0.444)
MIMT1; PEG3;ZIM2	19	Within 500 bp of TSS; 5′UTR; 5′UTR	Island	0.207	(0.088, 0.332)
CIRBP; C19orf24	19	Body; Within 1000 bp of TSS	Island	0.176	(0.097, 0.256)
SMG6; SRR	17	Within 500 bp of TSS; 5′UTR	Shore	0.125	(0.039, 0.214)
GPR12	13	5′UTR	Island	-0.095	(-0.161, -0.030)

For each significant locus, including RefSeq symbol for gene(s) in closest proximity, chromosome, UCSC annotated feature, relation to UCSC CpG island. Effect estimates were reported for sites for which β^IV from the two-stage least squares (TSLS) modeling the square-root arcsine beta-values were significant at the 0.05 α-level, adjusting for whether conception was planned. Without this variance stabilizing transformation, the effect estimates are unbiased, but inference will not be correct. For interpretability, effect estimates were calculated among the significant sites without the transformation and 95% confidence interval for 1000 bootstrapped replicates were reported.

**Table 2:** Biological process Gene Ontology (GO) enrichment among the 7 GO annotated genes in closest proximity to the loci significantly associated with RBC folate-level

**Gene ontology term**	**Number in significant subset**	**Number in all GO annotated genes analyzed**	**Odds ratio**
Regulation of dephosphorylation	1	16	133.373
Nuclear-transcribed mRNA catabolic process, nonsense-mediated decay	1	19	111.111
mRNA export from nucleus	1	21	99.980
Telomere maintenance	1	24	86.913
mRNA catabolic process	1	29	71.357
Nuclear export	1	45	45.336
Humoral immune response	1	67	30.158
RNA transport	1	67	30.158
RNA localization	1	69	29.265
Nucleobase, nucleoside, nucleotide and nucleic acid transport	1	79	25.487
G-protein coupled receptor protein signaling pathway	2	534	8.915
Homeostatic process	2	555	8.558

Significant over-representation of GO terms among these 7 genes relative to the 10,024 GO annotated genes included in the original analysis was assessed using a Hypergeometric distribution (p < 0.05).

**Figure 2:**
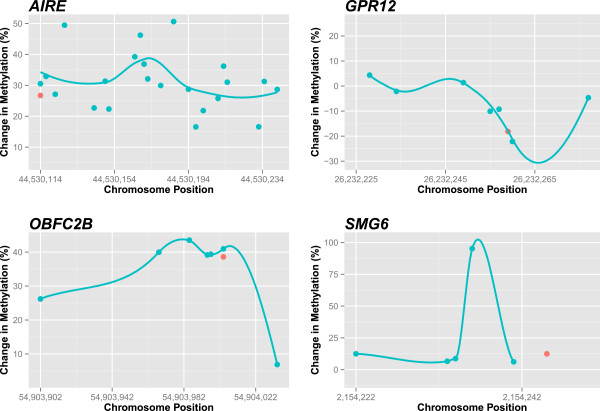
**Pyrosequencing the regions surrounding four of the most significant loci suggest site-specific changes reflect regional methylation variation associated with RBC folate; change in methylation level (%) per one unit increase log RBC folate estimated using MR among the loci assayed by pyrosequencing (blue) and on the microarray (red).** Regional changes were visualized by a loess curve of the site-specific estimates plotted against genomic location.

One drawback to the increased validity of the MR approach is the decreased precision of the estimates, precluding detection of significant results after accounting for multiple testing. While the strongest associations with folate detected using the MR approach did not meet genome-wide significance, we contend the decreased bias of the MR approach provides a more compelling ranking of loci for validation. Pyrosequencing the regions surrounding four of the most significant loci suggested these site-specific changes reflect regional methylation variation associated with RBC folate, corroborating this assumption (Figure 2).

**Figure 3:**
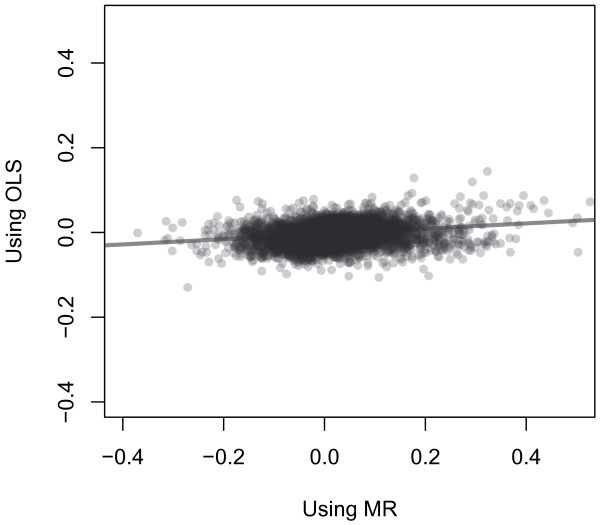
**The observed correlation (blue line) of the associations between methylation and log RBC folate levels, for the 16,989 loci estimated using the Mendelian Randomization and standard ordinary least squares methods.** Methylation level was square-root arcsine transformed to stabilize the variance. Models adjusted for conception intention.

Compared to the estimates obtained using TSLS approach, an appreciable effect of log RBC folate on methylation level would not have been detected using ordinary least squares (OLS), adjusting for conception intention (Figure 3). The range of TSLS estimates was nearly 4 times greater than that of the corresponding ordinary least squares values, spanning from -0.371 to 0.556, compared to -0.129 to 0.144. This increased range likely reflects both bias towards the null using the ordinary least squares approach and the decreased precision associated with the two-stage estimation. While the correlation between the parameter estimates was highly significant, it was very weak due to the disparity in effect magnitude (R^2^ = 0.194; p < 0.0001). Among the significant loci detected using the TSLS approach, only the locus within the 5′ UTR of GPR12 was one of the 10 most significant sites identified using the ordinary least squares approach (Tables 1 and 3). Besides this one site, none of the other 10 most significant sites identified using the ordinary least squares approach were within the 1000 most significant sites detected using the TSLS approach. Among this subset of CpG sites detected using the ordinary least squares approach, methylation level was only positively associated with log RBC folate at one site (Table 3). For all other sites within this subset, methylation level was inversely related to RBC folate level, which was in contrast to the TSLS parameter estimates. These results suggested that unmeasured confounding generally resulted in downward bias.

**Table 3:** Genes in proximity to the 10 loci with the most significant change in square-root arcsine methylation levels per one unit increase in log(RBC folate) using the ordinary least squares approach

**Symbol**	**Chr**	**UCSC annotated features**	**CpG Island**	**Δ β-value**	**P-value**
DMPK; DMWD	19	Within 1000 bp of TSS; 3′UTR; Enhancer	No	-0.078	0.000891
DAND5	19	Within 100 bp of TSS	No	-0.047	0.001093
TRIML1	4	1st Exon	No	-0.046	0.001603
GP9	3	Within 1000 bp of TSS	No	0.083	0.001762
POLL	10	Within 1500 bp of TSS	Shore	-0.031	0.001852
GCNT3	15	5′UTR	No	-0.049	0.001976
FUT2	19	Within 100 bp of TSS	Shore	-0.047	0.002012
GPR12	13	5′UTR	Island	-0.061	0.002106
FAM3D	3	Within 500 bp of TSS	No	-0.044	0.002132
COL16A1	1	5′UTR	Shore	-0.058	0.002294

For each significant locus, including RefSeq symbol for gene(s) in closest proximity, chromosome, UCSC annotated feature, and relation to UCSC CpG island. Models adjusted for conception intention.

**Figure 4:**
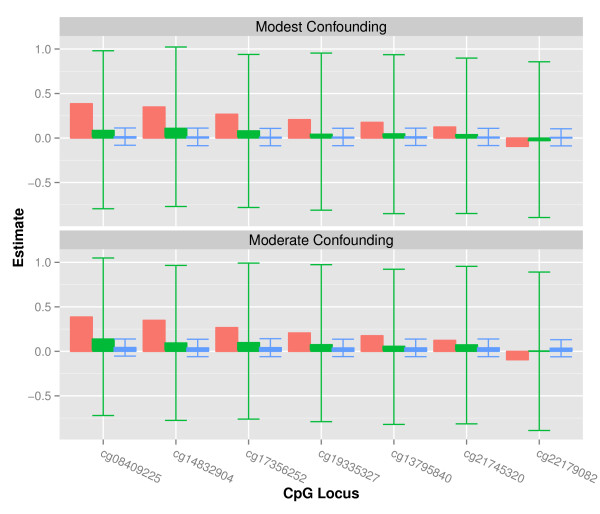
**Sensitivity of instrumental variable effect estimate (green) and ordinary least squares estimate (blue) to modest (*α***_***c***_ **=** ***β***_***c***_ **= 0.1) and moderate (*α***_***c***_ **=** ***β***_***c***_ **= 0.2) unmeasured confounding based on 10,000 simulations.** The red bars indicate the original TSLS estimates for the 7 significant loci modeled as the true causal effect in each simulation; the green and blue bars indicate the median instrumental variable and ordinary least squares effect estimates in the presence of unmeasured confounding. The error bars designate the 25th and 75th percentiles of these estimates.

Assuming the associations between log RBC folate and methylation level estimated using TSLS to be the true population parameters, the effect of varying degrees of confounding on observed estimates was assessed using both Mendelian Randomization and ordinary least squares. For these simulations, we utilized the parameters estimated by TSLS for the 7 loci significantly associated with log RBC folate. Compared to the ordinary least squares approach, Mendelian Randomization resulted in much more accurate but less precise estimates (Figure 4). Given a normally distributed unmeasured confounder with a modest positive association with both RBC folate and methylation, all observed effect estimates were biased towards the null. However, the Mendelian Randomization estimates were substantially more robust to this bias (Figure 4). For the ordinary least squares models, moderate unmeasured confounding completely removed the effect of log RBC folate on methylation (Figure 4). While the Mendelian Randomization estimates were biased in the direction of the observed confounded relations, they were consistently closer to the specified population parameter values than the parameters estimated by the ordinary least squares models. This simulation suggests that the decreased range of estimated associations between RBC folate and methylation level using the ordinary least squares approach may be at least partially attributable to residual cofounding.

## Conclusions

DNA methylation has become increasing integrated into public health studies as a modifiable indicator of the underlying biologic changes mediating the effects of endogenous and exogenous exposures on subsequent disease risk. However, the field of epigenetic epidemiology is still in its infancy and the effects of many exposures on methylation profile have yet to be explored and verified. In observational studies, the validity of estimated associations is always susceptible to criticism, given the possibility of residual or unmeasured confounding. Due to an array of factors that may influence both methylation and folate levels, inconsistencies among previously reported associations between maternal folate intake and neonate methylation patterns may be the result of bias. Using an instrumental variable approach, we were able to estimate the causal effect of cord RBC folate on DNA methylation level across the epigenome. While instrumental variable analysis also requires strong unverifiable assumptions, these assumptions are more cogent when genetic polymorphisms are employed as the instrument. Several papers have outlined the potential utility of a Mendelian Randomization approach in the context of epigenetic epidemiology [34,36]. These results demonstrate in practice, Mendelian Randomization can be a robust method to assess epigenetic modifications compared to the standard ordinary least squares approach. The cost of this improved internal validity is decreased precision. The reduction in power associated with the two-stage estimation precluded detection of significant changes in methylation level after correcting for multiple testing. By accounting for both measured and unmeasured common causes of exposure and outcome, we assert the loci with the most significant associations identified using the MR approach represent the most appropriate candidates for validation. Due to the use of a weak instrument, there is the possibility of residual bias in the direction of the confounded relationship [25]. However, simulation studies have demonstrated that even weak instruments substantially decrease the bias relative to the confounded association [25,51]. Our sensitivity analyses similarly illustrated this robustness in the context of epigenetic modifications. An additional drawback to this MR analysis is the possibility of over-fitting the data by estimating the association between the genetic variant and intermediate phenotype in the same cohort. To reduce this potential bias, future studies applying MR may consider a two-sample approach, using an external dataset to estimate the instrument-exposure association. However, the application of the two-sample approach is dependent on an additional, generally unverifiable assumption that the independent cohorts are drawn from the same underlying population. Building off the ‘Causality Equivalence Theorem’ presented by Chen [52], another recently suggested method to infer causal indirect effects of genotype on outcome relies on a series of models to statistically test necessary conditional independencies between covariates [53]. However, the application of this approach was deemed inappropriate given the associations between RBC folate and methylation level conditional on MTHFR genotype were likely influenced by common unmeasured causes of folate and methylation levels.

Although the Mendelian Randomization method provides valid estimates of the causal effect of RBC folate on methylation level across the genome, there is some uncertainty as to the functional implications. A majority of the significant loci were within the 5′ UTR of RefSeq annotated genes, while a few were located within the gene body or associated with a regulatory element, for which methylation may have disparate effects on transcriptional regulation. However, future studies can utilize these results to guide investigation of potential pathways mediating the influence of folate levels on developmental outcomes. These results should also be considered in the context of the samples analyzed, considering even closely related cell types, such as haematopoietic lineages, exhibit discrepancies in the methylome [54]. While this MR approach is robust to underlying disparities in cell type distribution that may influence both folate and methylation levels, it does not adjust for any downstream effects of RBC folate on cell type distribution that may impact observed changes in methylation. As a mediator, the analysis of the association between RBC folate and methylation is still valid without adjustment for cell type distribution. In this context, both fluctuations in cell type distribution and cell type-specific methylation may be meaningful, but these forms of variation are not partitioned by this analysis. Given this change in cell type distribution may be a component of the total effect RBC folate on methylation level and the inability to disambiguate the temporality of RBC folate and cell type distribution, we did not adjust for cell type distribution through recently proposed regression calibration techniques [54]. However, future studies of DNA methylation should be cognizant the potential impact of cell distribution when defining biologically meaningful variation given the specific research question.

Using a novel application of Mendelian Randomization methods to DNA methylation data, this study was able to provide insight into the biological mechanism mediating the effects of maternal folate on fetal development. An array of socioeconomic and culture factors influence individual diet and other environmental exposures that may also alter the epigenome. Given the many putative determinants of DNA methylation levels, there is a high likelihood of unmeasured confounding using standard regression techniques to assess the association between RBC folate and methylation levels. This study demonstrated that the amalgamation of these unmeasured predictors of folate and methylation level generally biases effect estimates towards the null. Using Mendelian Randomization methods it was possible to identify a significant, appreciable effect of RBC folate on methylation level of several CpG loci in cord blood that would have otherwise been obfuscated using standard regression techniques. Future studies will be able to use these results to guide additional analysis of the effects of periconceptional folate on candidate pathways. More generally, this study demonstrated the utility of Mendelian Randomization for the assessment of epigenetic modifications.

## Competing interests

The authors declare that they have no competing interests.

## Authors’ contributions

AMB conceived of the study and participated in its design, performed the statistical analysis, and drafted the manuscript. KBM participated in the study design and coordination and helped to review the manuscript. Both authors read and approved the final manuscript.
